# Anatomy, histology, and morphology of fish gills in relation to feeding habits: a comparative review of marine and freshwater species

**DOI:** 10.1186/s40850-025-00223-5

**Published:** 2025-02-07

**Authors:** Mohamed A. M. Alsafy, Hanan H. Abd-Elhafeez, Ahmed M. Rashwan, Atef Erasha, Safwat Ali, Samir A. A. El-Gendy

**Affiliations:** 1 Department of Anatomy and Embryology, Faculty of Veterinary Medicine, Alexandria University, Abis 10th P.O. 21944, Alexandria, Egypt; 2 Department of Cell and Tissues, Faculty of Veterinary Medicine, Assiut University, Assiut, 71526 Egypt; 3 Department of Anatomy and Embryology, Faculty of Veterinary Medicine, Damanhour University, Damanhour, 22511 Egypt; 4 Department of Life Science Frontiers, Center for iPS Cell Research and Application, Kyoto University, 53 Kawahara-cho, Shogoin, Sakyo-ku, Kyoto, 606-8507 Japan; 5 Department of Anatomy and Embryology, Faculty of Veterinary Medicine, University of Sadat City, Sadat City, 32897 Egypt; 6 Department of Anatomy and Embryology, Faculty of Veterinary Medicine, Minia University, Minia, 61519 Egypt

**Keywords:** Fish, Gill, Gill arch, Gill raker, Gill filaments

## Abstract

This systematic review highlights the similarities and variations in gill morphology, histology, and anatomical structure between differing fish species. The gill system consists of mainly four pairs of gill arches in most teleost fishes, such as sea bass, sea bream, grouper, and red porgy, etc., while it consists of three pairs of gill arches in pufferfish and striped-red mullet fish. However, *Clarias gariepinus* had five pairs, including an additional rudimentary fifth-gill arch. The gill structure consisted of gill arches, gill rakers, gill filaments, and secondary lamellae with varied shapes of gill arches such as hook, semilunar, L-shapes, and crescentic shapes. Each gill arch carried mainly two rows of gill rakers, lateral and medial, present in most teleost fishes (*Mugil cephalus, Boops boops, Pagrus pagrus, Sparus aurata*, European hake, Puffer fish, grey gurnard, sea bass, and sea bream). An additional row appears in *Clarias gariepinus* or two rows (accessory) in dusky grouper fish. The length and shape of gill rakers are mainly related to feeding habits. The gill rakers in lateral rows are longer, equal, or more in number and more developed than those of the medial rows, except at three gill arches in striped-red mullet fish, the second and third gill arches in pufferfish, and the fourth arch in *Pagrus pagrus*. gill rakers are absent at the first and second gill arches in *Bagrus bayad*. The gill arch carries additional structures, such as the air-breathing dendritic organ of the catfish, located in the suprabranchial chamber caudodorsal to the gills and composed of two main parts: small and large ones originated by main stems from the second and fourth-gill arches, respectively. The interbranchial septum can be smooth, form a median crest (seabream), or carry teeth or spines (seabass, pufferfish). Four transversely raised areas on each side are connected by transverse lines caudal to the base of the tongue (*Bagrus bayad*) and an elevated part at the level of the third-gill arch (*Tilapia zilli*). Scanning electron microscopy explained the micro-anatomical structures as varied shapes of pavement cells, mucus cell openings, taste buds on the gill arch, varied shapes of grooves or structures and spines near the gill filament side, varied shapes of gill rakers and their spines, and heights in varied feeding types of fish. Histological findings revealed various types of cells, such as superficial pavement cells, large chloride cells, mucous goblet cells, and basal epithelial cells. The lymph space is situated within the gill arch epithelia and is encompassed by cells that resemble tenocytes. The lymph space contains many types of immunological cells, including lymphocytes, granular leukocytes, and rodlet cells. The gill arch comprises sensory structures known as neuromasts and hyaline cartilaginous support. This review underscores the intricate relationship between gill structure and feeding habits across marine and freshwater fishes, highlighting the importance of understanding these variations for ecological, evolutionary, and aquacultural applications and feeding habits.

## Background

The gills form a highly characteristic feature of fishes, and their presence has a marked effect on the anatomy and functioning of the rest of the animal [1]. The gills play a significant role in adapting the fish to their environment. The primary functions of gills are gas exchange and waste elimination, and the gill epithelium also plays an osmoregulatory role [2–7]. Typically, gills are the gas exchange organs of water-breathing fishes, but in some species, they are also involved in gas exchange with the air [1, 8, 9].

The gills, like other systems in the fish body, undergo numerous modifications to adapt to the specific physicochemical conditions under which they must function [1]. There is a notable adaptation of fish to their environment and feeding habits, which is reflected in the morphological characteristics of their gills [4, 10–13]. There are many differences in the structural components of the gill arches, gill filaments, and gill rakers [3, 4, 12–14].

The intricacies of gill morphology have captivated researchers, prompting numerous studies that delve into the gill structures of various fish species [2–4, 9, 15–23]. The organization of gill filaments and gill rakers is closely related to the feeding habits of the fish [24–26]. The size and number of gill rakers control the size of food ingested; fish species with many and long gill rakers are typically filter feeders, whereas those with few and short gill rakers tend to be omnivores or carnivores [20, 27, 28].

The epithelial cells covering the gill arches, gill rakers, and gill filaments exhibit variable surface features in previously studied fish species, including the presence of micro ridges forming specific patterns, a mosaic of pavement cells, varied sizes of mucous and chloride cell pores, numerous pointed spines, and various types of taste buds [3, 4, 17, 29–31]

## Material and methods

A systematic review of the literature was conducted using three databases (Nusearch, CAB Abstracts, and PubMed), as well as ResearchGate, Google Scholar, and general internet searches. A lot of relevant papers were collected and evaluated for this review [32].

We selected figures from our unpublished data. We collected fish from the Mediterranean Sea, the Red Sea, and the Nile River. We prepared gross anatomy figures, scanning electron microscopy [33–36] and light microscopy slides with PAS stain, and semithin sections with toluidine blue stain, techniques according to [20, 37–41].

### Anatomy of gills

**Fig. 1:**
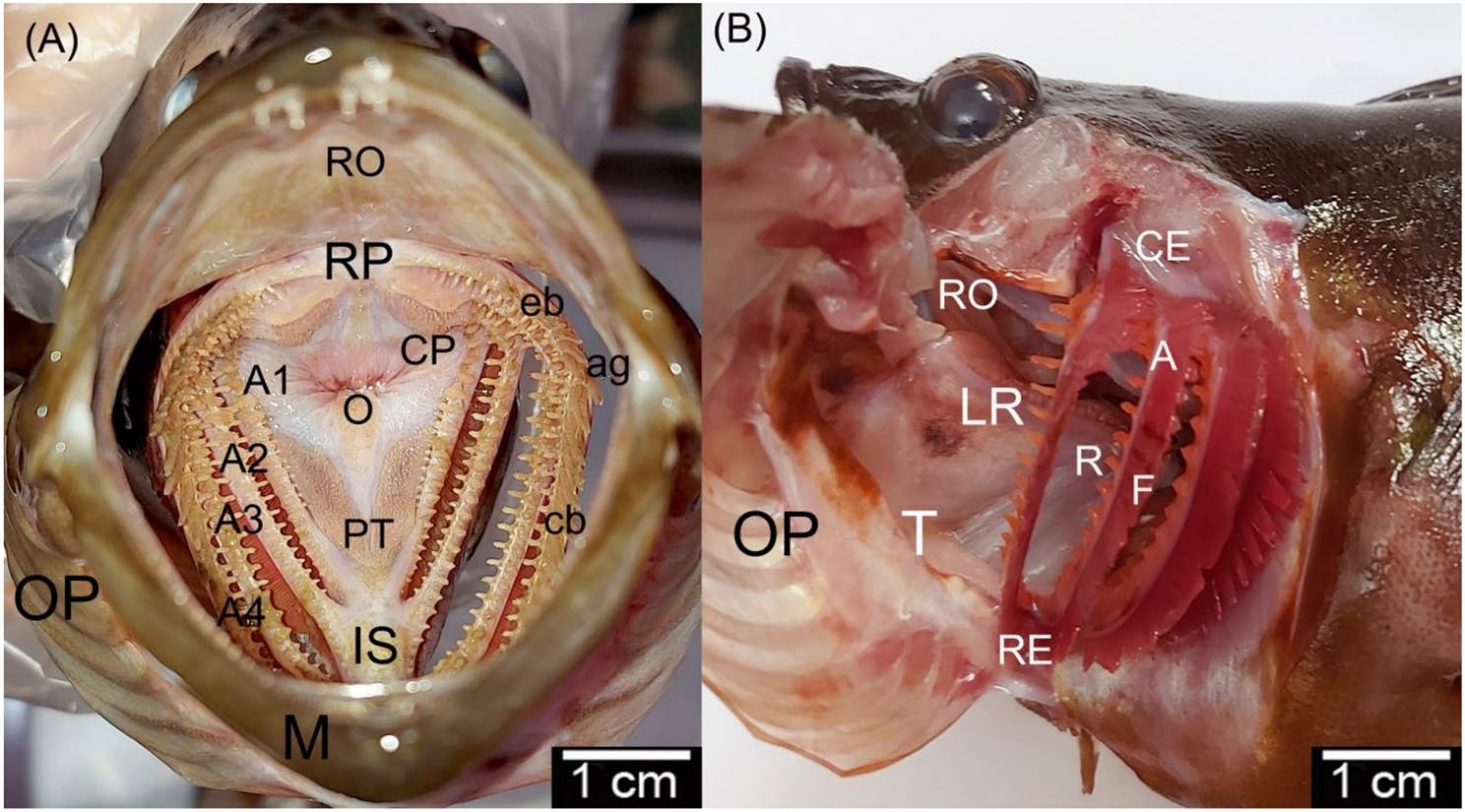
Gross images of the grouper fish: **A** rostral view of the opened mouth and **B** lateral view of the gills with reflected operculum that explained the structures and position of the gills. (A1, A2, A3, and A4) 1st–4th gill arches. rackers (R), a lateral row of gill rakers (LR), esophagus (O), interbranchial septum (IS), the mandible (M), the roof of the pharynx (RP), the roof of the oral cavity (RO), pharyngeal teeth (PT), chewing pads (Cp), operculum (OP), rostral extremities (RE), caudal extremities (CE), short epibranchial (eb), long ceratobranchial (cb), angle of epi-ceratobranchial union (ag), gill raker (R), gill arch (A), gill filaments (F), and tongue (T)

The gill system was confined within two interconnected gill chambers. The gill chambers were bounded ventrally by the mandible, dorsally by the roof of the oral and pharyngeal cavities, and laterally by the operculum. While medially, they were continuous with each other. Most gills of the teleost fishes’ were arranged on each side from lateral to medial as the first, second, third, and fourth gills [3, 4, 17]. In the dusky grouper [19], each gill was semilunar in shape, consisting of a gill arch that carried gill rakers on its concave border and gill filaments on its convex border (Fig. 1A and B).

Sea bream and sea bass are carnivorous fish that primarily feed on large-sized food items such as mollusks, small fish, crustaceans, insects, and decaying organic matter. Consequently, their gills are characterized by a prominent angle of curvature at the union of short epibranchials and long ceratobranchials from the first to fourth pairs of gill arches toward the dorsal side, similar to the carnivorous feeder catfish *Rita rita* [42] and *Eugerres brasilianus*, as well as the omnivorous feeder [31]. In contrast, gill arches in filter-feeding mullets, such as *Mugil cephalus*, show a lack of curvature or display an acute angle of curvature in the middle of the gill arches, indicating the degree of pharynx expansion [43].

#### Gill arch

**Fig. 2:**
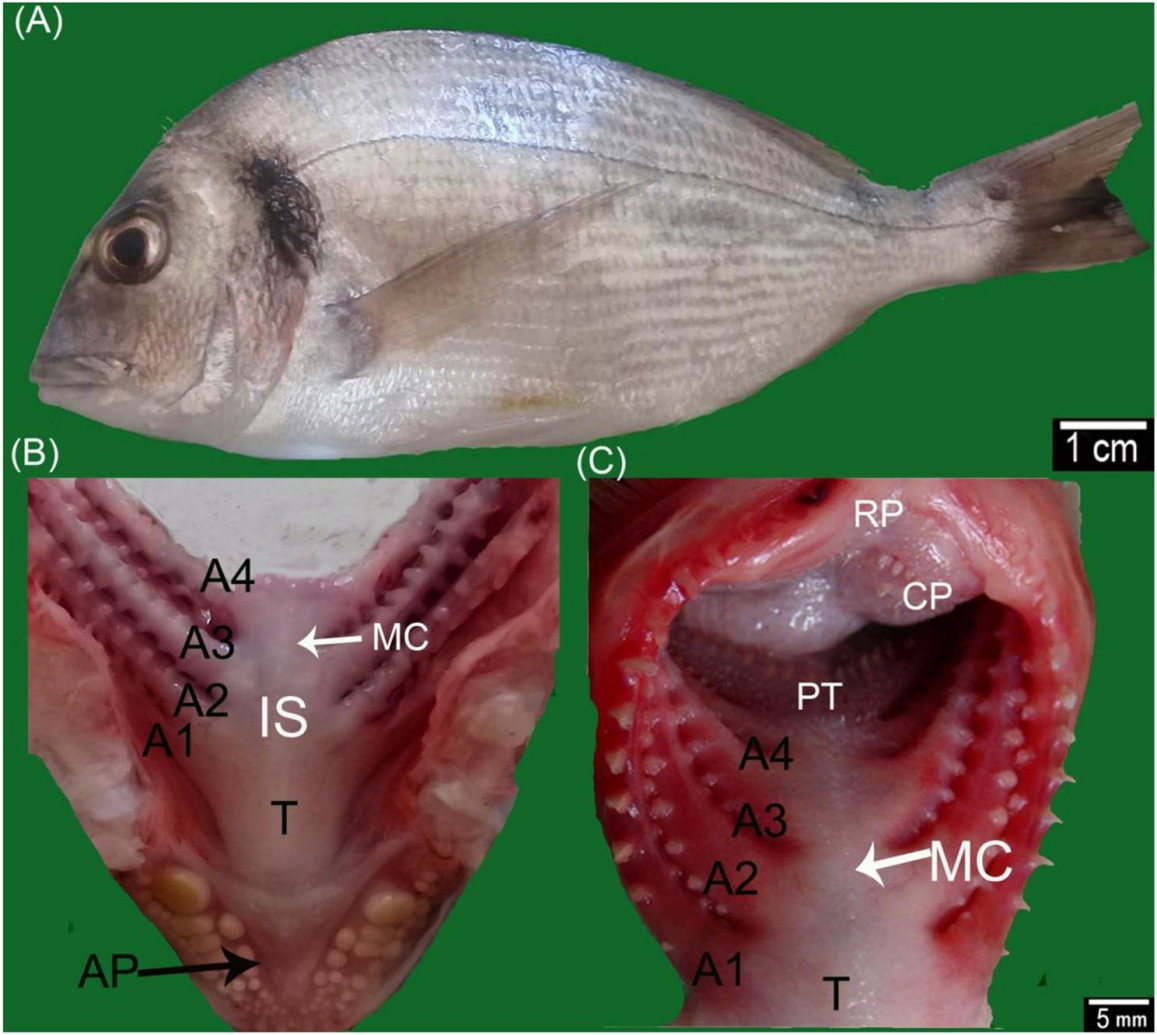
Gross images of sea bream fish: **A** lateral view of the fish and **B** and **C** the oropharyngeal cavity explained the following structures. (A1, A2, A3, and A4) 1st–4th gill arches, lateral row of gill rakers (LR), interbranchial septum (IS), roof of pharynx (RP), roof of the oral cavity (RO), pharyngeal teeth (PT), median crest (MC), chewing pads (Cp), tongue (T), and apical pouch (AP)

**Fig. 3:**
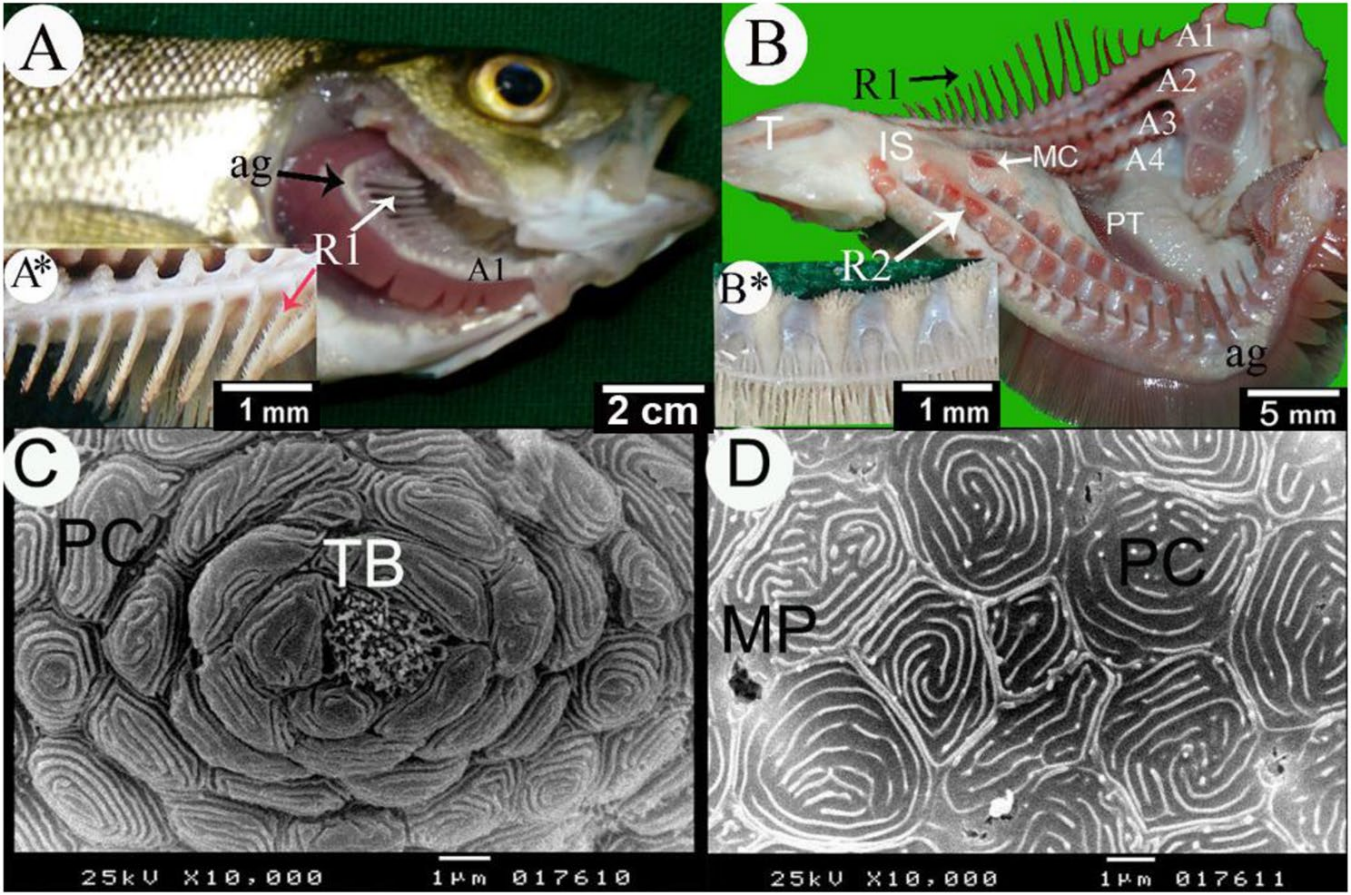
A, A^*^, B and B^*^ Gross images and **C** and **D** scanning electron micrographs of the gills of the Sea bass fish. **A** Lateral view of the gills after removing the operculum. **B** Rostral view to the gills chamber. **C**, and **D** SEM images of the 1st gill arch. (A1, A2, A3, and A4) 1st–4th gill arches, long gill rakers (R1), short gill rakers (R2), angle of epi-ceratobranchial union (ag), interbranchial septum (IS), median crest (MC), pharyngeal teeth (PT), pavement cells (PC), pores of mucous cell opening (P), tongue (T), spines (S), microplicae (MP), and taste buds (TB)

Each gill arch had two extremities: rostral and caudal. The rostral extremities of the gill arches were united, forming an interbranchial septum between the contralateral gills. The septum exhibited a median elevation like a crest in sea bream (Fig. 2A, B, C). At the same time, it appeared flattened dorsoventrally. It carried two rough, spiny small gill rakers at the level of the third gill in sea bass (Fig. 3A, B). The gills on both sides diverged caudolaterally, leaving a triangular area bounded rostrolaterally by the fourth pair of gills and occupied by the floor of the pharynx (Figs. 2B, C, 3A, B)—the caudal extremities of the four-gill arches curved dorsally, rostrally, and slightly ventrally. The gill arches were connected and attached to the operculum’s medial surface and the pharynx’s dorsolateral wall. The length and gaps between the four gill arches decreased medially, while the width and thickness of the four gill arches were similar. The gill arch was divided into a long ceratobranchial and a short epibranchial. The length ratio of the two parts varied from one gill arch to another. The union of the two parts of the gill arches toward the dorsal side showed a more prominent angle of curvature in sea bass than in sea bream [4].

The gill arch plays a crucial role in immunity [44]. The gill arches can effectively buffer the water flow pressure. The circular microridges can fix the mucus on the surface, and the strength of cells can be enhanced by the structure of circular microridges that can alleviate the mechanical damage of water flow. The gill arch taste buds have a role in food selectivity [19].

**Fig. 4:**
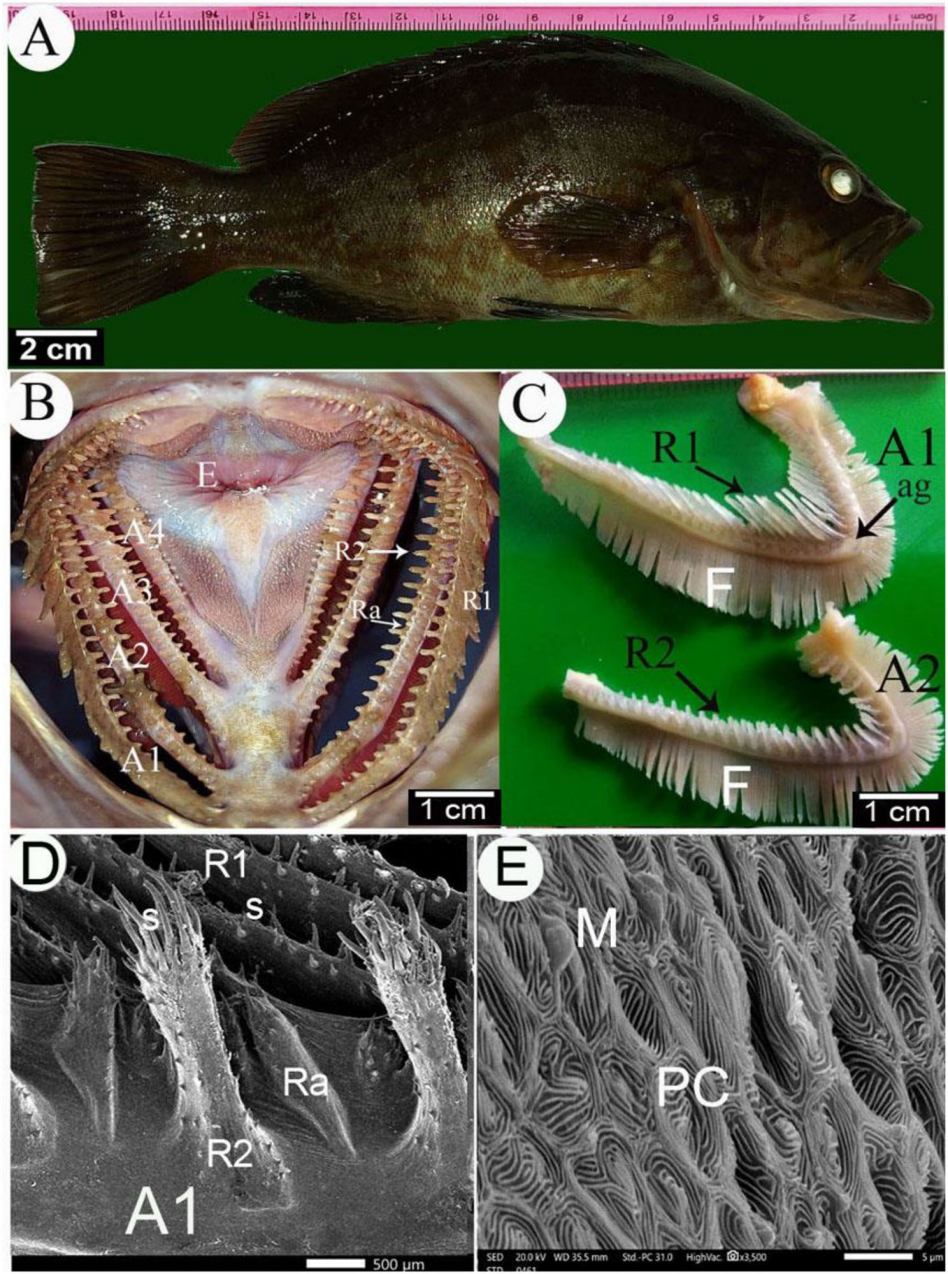
**A, B**, and **C** Gross images and **D** and **E** scanning electron micrographs of the gills of the dusky grouper fish. **A** Lateral view image of the dusky grouper fish. **B** Rostral view to the gills chamber. **C** Lateral view of the 1st and 2nd gill arch. **D** and **E** SEM images of the medial and dorsal view of the 1st gill arch. (A1, A2, A3, and A4) 1st–4th gill arches, long gill rakers (R1), short gill rakers (R2), accessory gill rakers (Ra), angle of epi-ceratobranchial union (ag), spines (S), solitary spines (So), pavement cells (PC), esophagus (E), microplicae (M), and gill filaments (F)

In the dusky grouper [19], the gill chamber had four gills on each side. They were semilunar in shape. The length of the gill arches from the 1st to the 4th gill arch were 5.27 cm, 4.2 cm, 3.2 cm, and 2.8 cm, respectively. Each gill arch had a long horizontal part. The ceratobranchial parts were four times the length of the short epibranchials, and the angle between the two parts of the gill was around 78° (Fig. 4A, B, C).

In Puffer fish [45], the gill arches had crescentic shapes and were directed rostroventrally. The rostral border of the gill arches formed a wide interbranchial septum that appeared quadrilateral and flattened dorsoventrally, carrying a line of small spiny gill rakers ventromedially to the third-gill arch. The caudal extremities of the three gill arches appeared curved dorsally. They were connected and attached to the dorsolateral wall of the pharynx. The lengths of the three gill arches were measured at 4.7 cm, 4.5 cm, and 4.1 cm, respectively, from lateral to medial. Each gill arch was divided into a long ceratobranchial and a short epibranchial part, demarcated by a slightly prominent angle. The length ratio of the two parts varied from one gill arch to another, decreasing from lateral to medial.

In grey gurnard fish [15], the gill arch had a crescentic shape. The rostral extremities of the gill arches united to form a wide interbranchial septum that appeared as a quadrilateral narrow structure carrying four transverse elevated crests.

In the striped-red mullet [15], the interbranchial septum had a median longitudinal elevated crest. The gill arches were connected and attached to the dorsolateral wall of the pharynx. Each gill arch lacked a distinct angle between the ceratobranchial and epibranchial parts, making it difficult to differentiate between them. The gill arches were connected rostrally and caudally.

In European hake [17], the thickness and width of the four-gill arches were similar, while the length and gaps between the gill arches decreased medially. The lengths of the four gill arches were measured at 5.3 cm, 4.7 cm, 4.5 cm, and 4 cm, respectively. Each gill arch had a long ceratobranchial part and a short epibranchial part. The length ratio of the two parts varied between the gill arches, with both parts demarcated by an angle, which was very narrow at the level of the second-gill arch.

In *Bagrus bayad* [3], the gill arch has two parts: a long ceratobranchial and a short epibranchial, clearly forming an angle between them in the first three gill arches, but not obvious in the fourth-gill arch. The gill arch was crescentic in shape and carried the gill rakers on the rostral concave border. The gill arches were attached rostrally to the mandible, forming a wide interbranchial septum consisting of four transverse raised areas on each side, connected by transverse lines caudal to the base of the tongue. The lengths of the gill arches were 7 cm, 5.5 cm, 5.5 cm, and 5 cm for the 1st, 2nd, 3rd, and 4th gill arches, respectively.

In the Nile River: *Oreochromis niloticus, Chrysichthys auratus*, and *Clarias gariepinus* [29], the gill system consisted of four pairs of gills. *Clarias gariepinus* also had a rudimentary fifth gill. Each gill was semilunar in shape, and the interbranchial septum was flattened dorsoventrally. The gills on both sides diverged caudolaterally, leaving a triangular-shaped area bounded rostrolaterally by the fourth pair of gills and occupied by the floor of the pharynx. This floor was modified into two distinct structures: the hypopharyngeal bone carrying the pharyngeal teeth and the lower pharyngeal jaw. The roof of the pharynx, opposite the lower pharyngeal jaw, was modified into an oval-shaped structure. The gaps between the gill arches were wider in *Clarias gariepinus* than in the other two species and narrower in *Oreochromis niloticus*.

In *Clarias gariepinus* [29, 46, 47], the breathing dendritic organ situated in the suprabranchial chamber caudodorsal to the gills. This organ consisted of small and large parts originating from the main stems of the second and fourth-gill arches. The size of the rostral small part was nearly half that of the large one, occupying the majority of the rostral compartment of the suprabranchial chamber. The large caudal part occupied most of the middle caudal compartments of the suprabranchial chamber. Both parts were connected to their corresponding gill arches by a cartilaginous joint. They originated from a small smooth surface main stem, divided into several secondary branches, ending in bulbous-like structures.

In *Tilapia zilli* [48], the gill system is located within two connected gill chambers. It was bounded by the mandible (ventrally), the operculum (laterally), the roof of the oral cavity (dorsally), and the base of the pectoral fin (caudally). The gill arches were crescentic in shape, carrying a row of gill filaments on their convex border and two rows of gill rakers on their concave border. The interbranchial septum was wide at the rostral part, quadrilateral in shape, with an elevated portion at the level of the third-gill arch. An angle in each gill arch divided it into a dorsal long ceratobranchial and a ventral short epibranchial part.

In common carp (*Cyprinus carpio*), the interbranchial septum connects the gills on both sides. The common carp’s gill has four gills on each side. It was arranged in a cranial-caudal direction. Each gill arch had two lateral and medial rows of gill rakers; the length of the gill arch decreased from the right first to the fourth gill arch (26.02 ± 2.04 mm, 24.69 ± 2.08 mm, 22.47 ± 1.19 mm, 19.35 ± 11.11 mm) [49].

#### Gill rakers

Gill rakers were cartilaginous or bony projections located on the gill arches that aid food collection and feeding habits [20]. their structure and shapes varying according to the feeding habits of the fish [50] (Fig. 4B, C). They were distributed unevenly across the branchial gill arches, typically concentrating in the first gill arch [51, 52]. Gill rakers served a dual function: they redirected water flow initially and then acted as a sieve, filtering food particles by trapping them with their mucous cover before ingestion [53].

Gill rakers are used to capture food and facilitate feeding behavior. Gill rakers varied in size and number among fish that consumed large prey. Plankton feeders possessed elongated, numerous, and diverse lamellae or ornamental gill rakers that functioned as effective sieves, allowing water to pass while trapping solid food particles [54, 55]. The number, shape, and spacing of gill rakers reflected the feeding behavior of fish species; fewer short gill rakers were found in carnivores and omnivores, whereas filter feeders exhibited numerous long gill rakers [4, 19, 52, 56–58]. Gill rakers are presented in small numbers in fish that consume large meals. Fish that only eat plankton have long and varied lamellae, or ornate gill rakers, that capture solid nutritional grains while allowing water to pass through during respiration [59]. Gill rakers are not suggested as a physical colander or strainer during filtration. However, it should be noted that the flow dynamics of liquids generate numerous vortices in the liquids received into the mouth, resulting in cross-flow filtration. The distance between the gill rakers is critical in removing particles from the suspension [60]. Furthermore, gill rakers are the anatomical part investigated in the discovery of new of new species [61], and they serve as a model for the industrial design of commercial filtration systems [60].

In carnivorous fish, the lateral gill rakers of the first-gill arch often bore small spines to prevent the escape of slippery prey, whereas subsequent rows had shorter gill rakers [4, 53, 62]. Anterior gill rakers on the first gill arch appeared as clusters of small tooth patches adapted for piscivorous feeding, while in herbivorous feeders, they resembled needle-like spines with secondary projections (Fig. 3A, A^⁎^). Detritivores and planktivores typically possessed the highest number and length of anterior gill rakers [63, 64].

European whitefish with sparse gill rakers tended to consume mollusks, crustaceans, and insect larvae, while those with denser gill rakers fed on zooplankton, chironomid pupae, and surface insects [63].

In species like sea bass, two types of gill rakers were observed: well-developed gill rakers with minute spines on the first gill arch and shorter gill rakers on subsequent gill arches [53, 62]. These gill rakers varied in length and structure across the gill arches, with sea bream displaying nearly parallel arrangements and sea bass exhibiting interdigitated patterns [4]. The gill rakers in sea bream were short and wide-based with sharp ends, whereas in sea bass, they were cylindrical with tapering ends and medial surfaces resembling saw blades, while lateral surfaces were smooth. The number of gill rakers decreased from the first to the fourth-gill arch in both species, with sea bream having 14 to 8 lateral and medial gill rakers, respectively, on successive gill arches, and sea bass having approximately 20, 18, 16, and 12 gill rakers on the first through fourth gill arches respectively [4].

Moreover, in *Chaca chaca*, unlike the gill arches of other fish species, there are no gill rakers. This adaptation is directly linked to the feeding behavior and dietary preferences of the species, as first reported by [12].

#### Marine water fish (Table 1)

In *Siganus luridus* [20], the gill arches were L-shaped. Each gill arch had two different forms of gill rakers with asymmetrical arrangements on most parts of the gill arches: spine-like gill rakers on the rostral side, which are bifid or trifid spines, and duck toe-shaped rakers on the caudal side. The mean number of gill rakers on the 4th-gill arches is 19, 17, 13, and 10 (Table 1). In *Boops boops* [20], the gill arches were semilunar in shape. The first gill arch carried conical gill rakers with pointed ends on the lateral side, while the gill rakers on the medial side of the 1st gill arch and the medial and lateral sides of the 2nd, 3rd, and 4th-gill arches were short and had spines. The mean number of gill rakers on each gill arch was 21, 16, 14, and 13 on the 1st to the 4th gill arches, respectively (Table 1).

**Table 1:** Marine water fish studies on varied fish families, feeding types, number and shapes of the gill arches and gill rakers, and the references supported that

Fish	Family	Feeding type	No. gill arches	Gill rakers number								Shapes of gill arches	Shapes of gill rakers	References
				1L	1M	2L	2M	3L	3M	4L	4M			
*Siganus luridus*	Siganidae	Planktivores	4 pairs	19 ± 0.32		17 ± 0.45		13 ± 0.45		10 ± 0.89		Bifid or trifid spines, or duck toe-shaped gill rakers.	L shape	[20]
*Boops boops*	Sparidae	Omnivorous	4 pairs	21 ± 0.89		16 ± 0.32		14 ± 0.32		13 ± 0.89		1L = long conical gill rakers with sharp lateral sides. 1M to 4M had short spiny gill rakers.	Semilunar	[20]
*Pagrus pagrus*	Sparidae	Carnivorous	4 pairs	13 ± 0.45		9 ± 0.32				6 ± 0.9	18 ± 0.4	Short, had fine-needle spinules covering the gill rakers’ top.	Hook	[20]
*Sea bass*	Serranidae	Carnivorous	4 pairs	29	26	25	22	17	14	11	9	1L = long saw-like, the rest was short and smooth.	Semilunar	[4]
*Sea bream = Sparus aurata*	Sparidae	Carnivorous	4 pairs	11	11	10	10	10	10	8	7	Short, wide-based processes with sharp ends and blunt surfaces.	Bow-like	[4]
												Short, conical in shape, adapted to carnivorous feeding.		[64]
Red Sea seabream (*Diplodus noct*)	Sparidae	Carnivorous	4 pairs									Short, conical in shape.	Semilunar	[64]
Dusky grouper	Serranidae	Carnivorous	4 pairs	28	28	27	27	19	18	17	17	1L = 1ong cone shape, while the reset of gill rakers was cylindrical, short gill rakers.	Semilunar	[19]
				AC 27		AC 26		AC 17		AC 13		The accessory rakers were like the short gill rakers but smaller in length.		
European hake	*Merlucciidae*	Carnivorous	4 pairs	10	7	7	4	5	4	3	2	1L was triangular with pointed ends carrying spines, with the rest of the gill rakers elevation with blunt ends carrying spines.	———	[17]
Grey Gurnard Fish	Triglidae	Carnivorous	4 pairs	16	17	14	14	12	12	11	11	Long lateral and medial short gill rakers on the first gill arch, while the rest gill arches show short lateral and medial gill rakers in the grey gurnard.	Crescentic-shape	[15]
Striped Red Mullet Fish	Mullidae	Omnivorous	3 pairs	11	12	9	10	8	11	-	-	All three-gill arches were carrying short lateral and medial gill rakers.	Crescentic-shape	[15]
Puffer Fish	Tetrodontidae	Omnivorous	3 pairs	17	17	12	15	11	12	-	-	The gill arch has two rows of small lateral and slightly larger medial gill rakers.	Somewhat crescentic -shape	[45]
*Mugil cephalus*	Mugilidae	Detritus feeder	4 pairs									The lateral row had long and great numbers of gill rakers, while there were short and fewer numbers on the medial one.	Crescentic -shaped	[64]

In *Pagrus pagrus* [20], the gill arches look like a hook and carry short gill rakers with fine-needle spinules covering the gill rakers’ top, arranged in medial and lateral rows. The gill rakers of the first three gill arches are parallel, while on the 4th-gill arch, the gill rakers of the lateral row correspond to only three small gill rakers on the medial row. The mean number of gill rakers is 13, 9, and 9 on the lateral and medial sides of the 1st, 2nd, and 3rd-gill arches, respectively, while on the 4th-gill arch, the lateral gill rakers are 6, and the medial row has 18 gill rakers (Table 1).

In Sparidae (*Sparus aurata* and *Boops boops*) [64], the gill arch was bow-shaped with long and more developed gill rakers in the first row and short, less developed gill rakers in the second row. The gill rakers in the anterior row of the first-gill arch in most species of the family Sparidae were short, conical in shape, and elongated thick strips with slightly pointed ends and triangular bases in *Boops boops*.

In European hake [17], the first-gill arch had long lateral gill rakers that appeared triangular with pointed ends and carry spines, while the rest of the gill rakers in the row were short with blunt ends and carry spines. The number of gill rakers on the lateral side of the four gill arches is 10, 7, 5, and 3 on the first, second, third, and fourth-gill arches, respectively, while the number of medial gill rakers is 7, 6, 4, and 2, respectively. The gill raker length decreases from the first to the fourth gills (Table 1).

In Puffer fish [45], the gill rakers are located on the concave internal side of the gill arches. Grossly, each gill arch has two rows of gill rakers: small lateral gill rakers (1 mm) and slightly larger medial gill rakers (2 mm). The gill rakers of adjacent gill arches are interdigitated. The number of gill rakers on the lateral rows of the 1st, 2nd, and 3rd gill arches is 17, 12, and 11, respectively, while the number on the medial rows is 17, 15, and 12, respectively (Table 1).

In grey gurnard [15], each gill arch carried two rows of gill rakers: long lateral and short medial gill rakers on the first gill arch, while the rest of the gill arches showed short lateral and medial gill rakers. In the striped-red mullet, all three-gill arches carry short lateral and medial gill rakers. The gill rakers of adjacent gill arches are interdigitated. The average number of gill rakers varies slightly among gills in grey gurnard and striped-red mullet (Table 1).

In *Mugil cephalus* [64], the lateral row has long and numerous gill rakers, while the medial row has shorter and fewer gill rakers (Table 1).

#### Freshwater fish (Table 2)

In *Bagrus bayad* [3], the first two gill arches had only lateral rakers. The first gill arch carried well-developed long gill rakers, while the gill rakers on the subsequent rows were short. Most gill rakers were short, an adaptation to the carnivorous feeding habits of *Bagrus bayad* (Table 2).

**Table 2:** Freshwater fish studies on varied fish families, feeding types, number of the gill arches and gill raker, and the references supported that

Fish	Family	Feeding type	No. of gill arches	Gill rakers characteristics and another characteristic features	References
*Oreochromis niloticus*	Cichlidae	Herbivorous	4	Semilunar in shape, the gaps between the gill arches were generally narrow. The gill rakers of the medial row were directed dorso-medially, while those of the lateral row were directed dorso-laterally. The gill rakers appeared as relatively short and wide-based processes with tuberous ends.	[29]
*Chrysichthys auratus*	Claroteidae	Omnivorous	4	The gill arch was semilunar in shape.	[29]
				The gill rakers had two rows; the medial row was directed ventro-medially, while the lateral row was directed ventro-laterally. The gill rakers appeared as relatively short and broad-based processes with segmented tuberous ends.	
*Clarias gariepinus*	Clariidae	Omnivorous	4 and additional 5th rudimentary	branched bulbous dendritic structures originating from the second and fourth-gill arches. The gill rakers were presented in three rows: medial, intermediate, and lateral. The medial and lateral row gill rakers were numerous with long processes, while those of the intermediate row were few and short. The medial row was found only in the third and fourth gills, while the intermediate row was found in the four main gills,	[29, 46]
*Bagrus bayad*	Bagridae family	Carnivore	4	Absent medial rows of gill rakers at the first and second gill arches, the number of gill rakers ranged from 14 to 16. The first gill arch carried well-developed long gill rackers, while the gill rackers on the following rows were short. Crescentic outlines with a wide interbranchial septum,	[3]
Nile tilapia	Cichlid	Changed to Omnivorous from herbivorous with an increase in size	4	The length of the gill rakers increased from the first to the fourth gill. The number of the gill rakers was variable in the different gills: they were 28 in the first gill, decreased to (25) in the second and third gills, but increased again to reach a maximum value (38) in the fourth gill.	[29, 46]
*Tilapia Zilli*, redbelly tilapia	Cichlid	Herbivorous	4	Each gill arch carried two rows of the small, short, wide-spaced gill rakers: lateral and medial. The gill rakers on each row were nearly the same in size, except the lateral gill rakers of the first-gill arch were longer. The gill rakers of the adjacent gill arches were interdigitated with each other. The lateral gill rakers on each gill arch were directed dorsolaterally, while the medial ones were directed dorsomedially.	[48]
*Common carp (Cyprinus carpio)*	Cyprinidae	Omnivorous	4	The medial edges of four gill arches with zippers have the potential to interlock. A significant reduction in gill arch length was observed from the first to the fourth, with the second and third gill arches having the greatest number of gill rakers (2nd (25.38 to 29.00) and 3rd (25.87 to 28.62). The length of gill raker increases from the first to the fourth gill arch.	[49]

In *Tilapia zillii* [48], the gill arch carried two rows of small, short, wide-spaced gill rakers: lateral and medial. The gill rakers on each row were nearly the same size except for the lateral gill rakers of the first-gill arch, which were longer than the medial ones. Conversely, the medial gill rakers of the fourth-gill arch were longer than the lateral ones. The gill rakers of the adjacent gill arches interdigitated with each other. The lateral gill rakers on each gill arch were directed dorsolaterally, while the medial ones were directed dorsomedially (Table 2).

In *Oreochromis niloticus* [29], the gaps between the gill arches are generally narrow. The gill rakers of the medial row were directed dorsomedially, while those of the lateral row were directed dorsolaterally. The gill rakers on the same gill decreased in size towards the epibranchial part. They appeared relatively short and wide-based with tuberous ends. In *Chrysichthys auratus* [29], the gill rakers had two rows; the medial row was directed ventromedially, while the lateral row was directed ventrolaterally. The gill rakers appeared relatively short and broad-based with segmented tuberous ends (Table 2).

In *Clarias gariepinus* [29, 46], the gill rakers were in three rows: medial, intermediate, and lateral. The medial and lateral row gill rakers were numerous with long processes, while those of the intermediate row were few and short. The medial row was found only in the third and fourth gills, while the intermediate row was in all four main gills. The number of gill rakers in the intermediate row was 20, 23, 19, and 18 in the first, second, third, and fourth gills, respectively (Table 2).

In common carp, the number of medial row gill rakers from the first to the fourth gill arch at the right gill was (25.50 ± 2.45, 27.50 ± 2.78, 27.37 ± 2.61, 21.62 ± 2.00), while their lengths were (1.80 ± 0.18 mm, 2.15 ± 0.34 mm, 2.38 ± 0.30 mm, and 2.47 ± 0.28 mm) [49].

#### Gill filaments

Each gill arch carried double rows of well-developed and compactly arranged gill filaments. Each gill filament row was called a hemibranch, and the two hemibranchs together formed a holobranch. The hemibranchs of the four-gill arches were numbered in a lateromedial direction. The gill filaments appeared long in the middle and shortened toward the extremities [4]. The effectiveness of fish gills in extracting oxygen from water, as well as their adaptation to water and immunity, was reported by [4, 65].

### Scanning electron microscopy of gills

#### Gill arch

All surfaces of the gill arch are covered with a mosaic of irregular polygonal pavement cells with apparent, concentrically arranged surface ridges [4]. Microridges, resembling fingerprints, covered the exposed surfaces of the epithelial cells. The mucous pores varied in size, appearing as narrow, deep, rounded-to-oval holes with little or no visible internal structure. Ovoid grooves were observed on the dorsal third of the two gill arch surfaces between the roots of the gill rakers, showing short, sharp, pointed, curved spines protruding through the gill epithelium of sea bass (Fig. 3C, D). Taste buds were seen between the gill rakers on the gill arch surface, each distinguished by closely packed sensory protrusions towards the surface (Fig. 3C, D).

In the dusky grouper [19], the upper third of the gill arch height contained a longitudinal band with many wavy folds, irregular mosaic patterns, and polygonal epithelial cells. The surface of the gill arch between the gill rakers bases had many small spines and taste buds. On the dorsal surface of the gill arches, there was a circular group of spines, ranging from 5–8 spines with medially curved ends, located medially to the base of the main gill rakers or between them (Fig. 4D, E). Each spine had a pointed arrow cape-like structure on its apex, flanked distally with an annular groove, and the bases of the spines had an annular groove. The surface of the gill arch had a stone-like background texture.

The surface ultrastructure of gill arches and gill rakers was derived from studies on fish species having different feeding habits: *Rhinomugil corsula* [66], *Gadusia chapra* [67], filter feeder *Brevoortia tyrannus* [68], *Hypostomus commersonii* [69], *Prochilodus scorfa* [70], *Mugil curema, Mugil liza and Mugil platanus* [31], omnivorous *Fundulus heteroclitus* [43], *Cyprinus carpio* [71], carnivorous *Anabas testudineus* [66], *Notopterous chitala* [67], *Eugerres brasilianus* [72], *Cathorops strigosa* [73], carnivorous catfish, *Rita rita* [42], *argentinian silverside* [74], *Sakhalin trout* [75], snow *Trout Schizothorax* [76], and Indian major carp *Cirrhinus mrigala* [77].

The surface ultrastructure of the gill arches and the gill rakers of an herbivorous fish, the Indian major carp *Cirrhinus mrigala,* have closely lying short gill rakers and narrow inter-gill rakers channels on the gill arches, which were associated with filtering and retaining food particles [77]. The surface of the gill arch in the grey gurnard was characterized by gill rakers with multiple small spines. In the striped-red mullet, the surfaces of the gill arch appeared smooth, except for a region with many longitudinal micro ridges demarcating the area between the gill rakers and the origin of the gill filaments [15]. Additionally, the striped-red mullet had many taste buds on the smooth surface of the gill arch.

In the European hake [17], the surface of the gill arches was wrinkled in some areas. The lateral and medial surfaces of the first gill arch differed from those of the other gill arches. The first-gill arch had a longitudinal line of spines presented in groups, forming circular, cuboidal, rectangular, oval, and triangular shapes with spaces between them. In *Bagrus bayad* [3], the gill arches are entirely covered by pavement cells. The pavement cells had circular and oval openings of variable sizes without internal structures. There were two types of pores: chloride and mucus cells with their secretions.

In *Tilapia zillii* [48], the middle part of the gill rakers did not have any characteristic structures but had two different elevated structures near the gill filament: an oval-shaped leaf-like structure and a rounded-shaped structure. The elevated oval leaf-like structure had a round end carrying two lateral rows of triangular pointed spines separated by a median groove, which had a few type I taste buds. The elevated, rounded-shaped structure consisted of two round-shaped structures connected to each other. The surface of the four-gill arches was covered with a mosaic of irregular polygonal pavement cells, with numerous pores of different sizes for chloride and mucous cells. Type I taste buds were present on the gill arch.

In three types of fishes from the Nile River (*Oreochromis niloticus, Chrysichthys auratus,* and *Clarias gariepinus)*, the epithelium covering the gill arches had a mosaic of variable dimensions [29]. The exposed surfaces of the epithelial cells were covered by micro ridges, which were straight and compactly arranged in *Oreochromis niloticus* but parallel to each other or irregularly interwoven to form web-like patterns in *Chrysichthys auratus* and *Clarias gariepinus*. Mucous cells were scattered among the gill arch epithelial cells, being more numerous in *Chrysichthys auratus* and *Clarias gariepinus* than in *Oreochromis niloticus*. These mucous cells were often filled with blobs of mucous secretion.

In common carp [49], a median crest is demonstrated on the gill arches between the lateral and medial gill rakers. The interbranchial septum had mucosal folds that ran from cranial to caudal and finger-like papillae near the pharyngeal teeth and replaced mucosal folds. Type II and III taste papillae were found in the anterior pharynx.

#### Gill rakers

In sea bream [4], the gill rakers appeared as short, wide-based processes with sharp ends and blunt surfaces. Many sharp-pointed spines protruded on the gill rakers’ ventral border, while the dorsal border was free from spines. In sea bass, the first gill arch had long cylindrical gill rakers with tapering ends. The medial surface of these gill rakers carried conical spines of different sizes and directions, while the lateral surface was smooth. The second type of gill rakers showed a short cylindrical mass of spines surrounded by a deep, circular groove. Taste buds were noticed at the summit of the epithelial protuberances, at various elevations between the spines on the second type of gill rakers.

In the dusky grouper [19], each gill arch had two main rows of gill rakers (lateral and medial) and two accessory rows of gill rakers arranged alternately with the medial and lateral gill rakers. The two main rows were nearly similar in length on the second, third, and fourth-gill arches, while on the first gill arch, the lateral row had long gill rakers, and the medial row had short gill rakers. Spines were especially prominent on the long gill rakers. The lateral gill rakers of the first gill arch had a long cone-shaped part, smooth on the lateral surface and spined on the medial surface, carrying three types of spines: long, medium, and short. The small spines on the medial aspect of the lateral gill rakers were surrounded by an annular groove, with taste buds and mucous pores arranged in a linear state. The medial row of the first gill arch and the rows of the following gill arches had cylindrical, short gill rakers. The base of the medial gill rakers of the first gill arch was circular. In contrast, the gill rakers’ bases of the medial and lateral gill rakers of the following gill arches were compressed cylindrical with a dorsal extension on the gill arch. The short gill rakers had small spines on their bases and bodies, with moderate and long spines emerging from the epithelial covering. The short spines had small openings at their bases with a wavy epithelial covering and taste buds’ protrusions, while taste buds III were sunken in the epithelium with sensory protrusions. The accessory gill rakers resembled the short gill rakers but were smaller and had three types of spines. The three types of spines were measured, with the long spine on the apex of the short gill rakers being longer than the long spines of the long lateral gill rakers of the first gill arch (Fig. 4D).

The gill rakers of *Siganus luridus* were relatively smooth, ending in spine-like structures with different shapes: single spine, bifid, trifid, and quadrate, resembling duck toes, which act as filters to catch algae particles. Adapted to vegetarian feeding, *Siganus luridus* progresses from feeding on zoo and phytoplankton as larvae to finer algae as adults [64, 78]. They primarily consume algae (99.73%), seagrass, and rubble [79]. For herbivorous fish, the gill rakers were mainly short, acting as branchial sieves to efficiently filter small food particles from the water [77]. The herbivorous black fish’s gill rakers direct water toward the oral cavity roof, where the mucous covering traps food particles before being ingested [80]. It is suggested that gill rakers perform a dual function: changing the direction of the water and filtering food particles [80].

*Boops boops* had long gill rakers appearing conical with pointed ends on the medial and lateral sides of the first-gill arch. The following gill arches had long gill rakers on their medial sides and short gill rakers on their lateral sides. All gill rakers carried various shapes of spines. These long and short-spined gill rakers, and the narrow spaces between them, are specialized for different types of food particles, as *Boops boops* are omnivorous [53, 64, 81, 82]. The long gill rakers were semi-conical with tapered ends, and their medial surfaces had needle-like spines adapted for sorting plankton, similar to observations by [64]. Additionally, wedge-shaped spines at the base of gill rakers act to increase the seizing of slippery and smooth prey [78].

The gill rakers of *Pagrus pagrus* were short with fine-needle spines covering their tops. The height of the gill rakers gradually decreased from the first to the fourth-gill arch. The spines were conical with pointed, curved, or straight ends, confirming the carnivorous nature of *Sparidae* fish [53, 64, 83]. *Pagrus pagrus* had a low number of gill rakers but a larger number in the last row, which may increase the seizing of slippery, smooth, and slimy prey [62].

The arrangement of gill rakers in medial and lateral rows in *Pagrus pagrus, Boops boops*, and *Siganus luridus* plays a crucial role in their feeding habits. The equal number of gill rakers on the medial and lateral rows in all gill arches, except in the last gill raker row of *Pagrus pagrus*, is a key factor in their feeding strategy. The highest number of gill rakers on the first gill arch, particularly in *Boops boops, Siganus luridus*, and *Pagrus pagrus*, enhances cross-flow filtering and limits the escape possibilities of small prey [52, 64, 84, 85]. The relationship between prey size and the gill raker gap and standard length is a fascinating correlation that underscores the importance of gill raker characteristics in the feeding habits of fish species [86]. The herbivorous *Siganus luridus* had many gill rakers and narrow spaces between them. In contrast, the carnivorous *Pagrus pagrus* had fewer gill rakers and wider spaces, and the omnivorous *Boops boops* had an intermediate range. This indicates that herbivorous species prefer smaller food particles, while carnivorous and omnivorous species prefer larger food particles.

Short and tuberous gill rakers in *Oreochromis niloticus* were effective filters of food. In *Chrysichthys auratus*, gill rakers were short with a broad base, which strain water to bathe the gills and prevent solid particles from passing over them. Gill rakers in *Clarias gariepinus* were long, cylindrical, and arose at acute angles to the gill arch, helping to strain food and other materials, thus protecting gill filaments from damage [29].

The gill rakers and spine distribution on the first gill arch of the European hake differed from that of the other three gill arches on the lateral and medial surfaces [17]. In *Tilapia zillii* [48], scanning electron microscopy showed the gill rakers on both rows were short and small. Each gill raker had a median central axis surrounded by two lobulated lateral regions. The median central axis was smooth with a blunted round end, and, under higher magnification, its surface was covered with a mosaic of polygonal pavement cells with clear, concentrically arranged surface cell ridges, giving them a fingerprint appearance. Oval pores of the chloride cells and a few small-sized taste buds type I were present. The two lobulated lateral areas carried numerous pointed spines, taste buds, and a few chloride cell pores.

In common carp [49], the lateral and medial gill rakers had different numbers of dome-like projections on the side facing the oral cavity. Each gill raker also ends in a conical tip. The surface epithelium was composed of irregular, polygonal-shaped cells. Microridges were found to be arranged differently on cells. Two microridges were discovered: elongated microridges and short microvillus-like.

#### Gill filaments

The gill filaments carried leaf-like lamellae that arose from both sides. Under higher magnification, the surface of the gill filaments and lamellae was covered by a mosaic of irregular, polygonal epithelial cells. These cells exhibited many concentric microplicae, which appeared large at the bases of the lamellae. Numerous large, rounded to oval openings of chloride cells with projections, as well as smaller openings of mucous cells, were observed [4].

In the dusky grouper [19], each gill arch carried two rows of gill filaments on its ventral side. The gill filaments were measured at three levels (middle and two extremities of each gill arch), showing that the gill filaments were longest at the middle of the gill arch and shortest at the rostral end. The bases of the gill filaments exhibited irregular folds with stony-like projections. The primary gill filaments had secondary lamellae that contained the pores of mucous and chloride cells. The mucous pores were larger in diameter than the chloride cell pores. The secondary lamellae had pavement cells on their surfaces, with fingerprint-like epithelial coverings and characteristic wavy folds.

On the surface of the gill filaments of the European hake, longitudinal ridges were present, carrying pores of chloride and mucous cells [17].

### Microridges and sensory organs

All surfaces of the gill arch are covered with microridges in teleost fish, resembling fingerprints, covered the exposed surfaces of the epithelial cells [44]. The gill arch has circular microridges can fix the mucus on the surface, and the strength of cells can be enhanced by the structure of circular microridges that can alleviate the mechanical damage of water flow. Microridges were found to be arranged differently on cells. Two microridges were discovered: elongated microridges and short microvillus-like in common carp [49].

The surface of the gill arches, gill rakers, and gill filaments exhibit various types of taste buds [3, 4, 17, 29–31]. The gill arch taste buds have a role in food selectivity [19]. Taste buds were seen between the gill rakers on the gill arch surface, each distinguished by closely packed sensory protrusions towards the surface in see bass (Fig. 3C, D). Additionally, the striped-red mullet had many taste buds on the smooth surface of the gill arch [31]. In *Tilapia zillii* [48], a few small-sized type I taste buds were present on the gill arch. In sea bream [4], the taste buds were noticed at the summit of the epithelial protuberances, at various elevations between the spines on the second type of gill rakers. In the dusky grouper [19], The small spines on the medial aspect of the lateral gill rakers were surrounded by an annular groove, with type I taste buds, while taste buds III were sunken in the epithelium with sensory protrusions.

In general, the presence of taste buds on the gill filaments has not been reported in any fish species. However, a recent study by [13] documented the presence of taste buds on the gill filaments of the Indian Moth catfish, *Hara hara*, for the first time. They associated this adaptation with the food and feeding habits of the fish.

### Light microscopy of gills

**Fig. 5:**
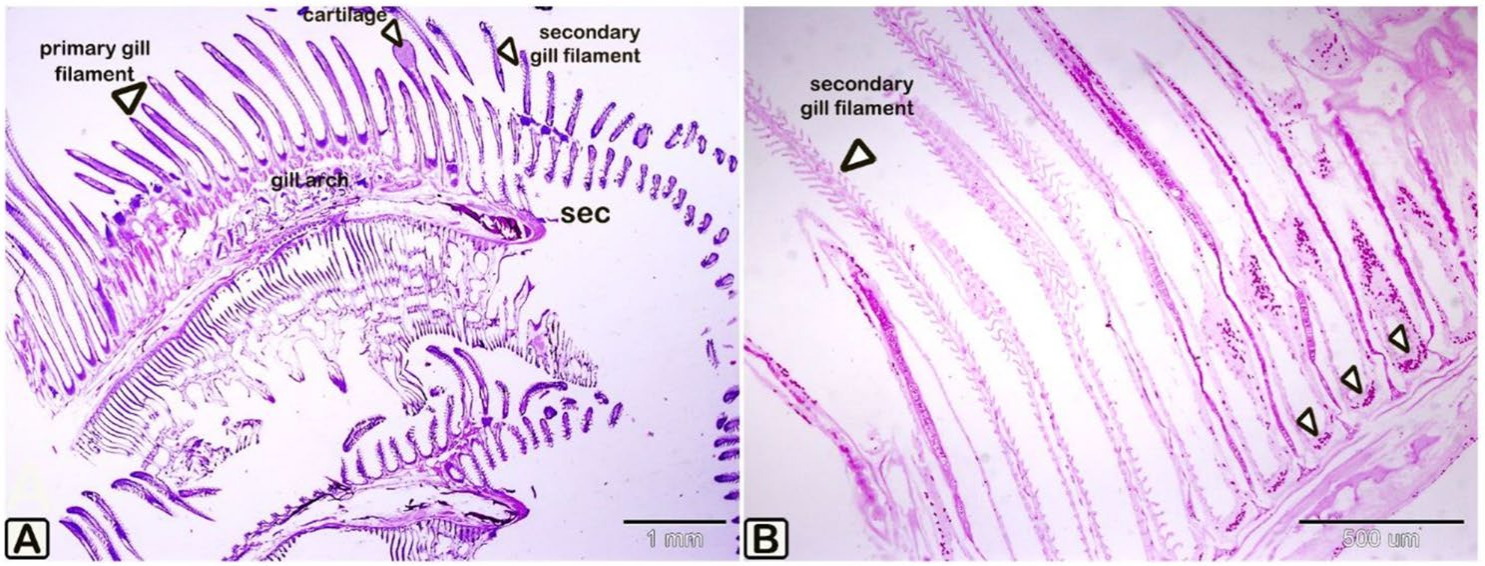
The paraffin sections of the gills of the silver carp stained by HE stains (**A**) and PAS stain (**B**). The gills consist of gill arch, gill filament (primary gill lamellae, arrowheads) and the secondary gill lamellae(arrowheads). Note arrow heads on figure B pointed out to rodlet cells

**Fig. 6:**
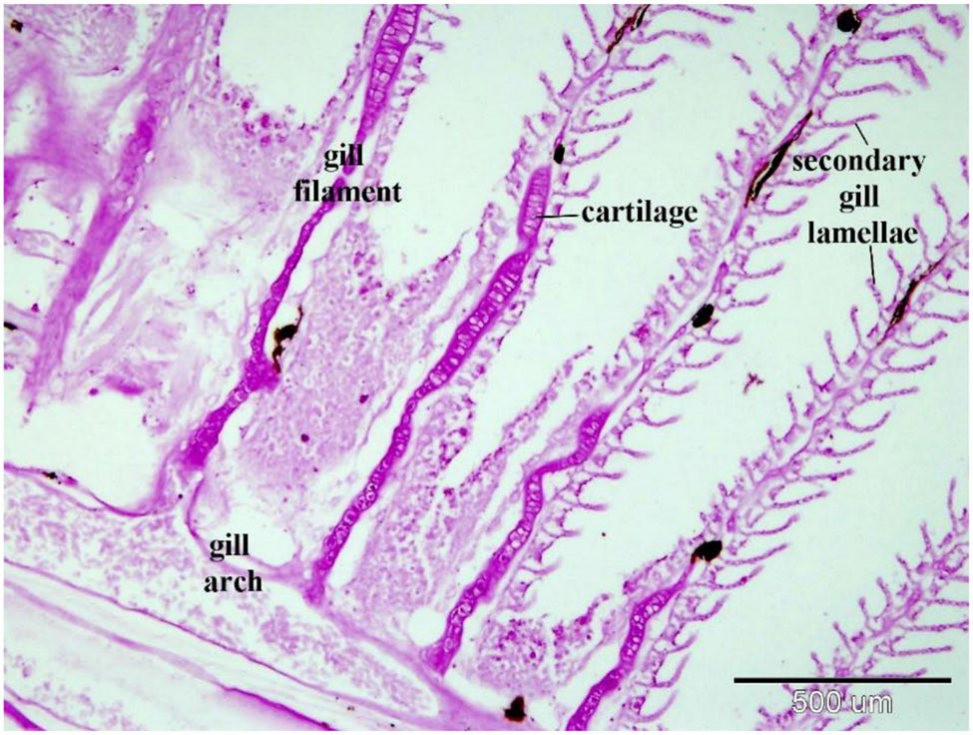
A paraffin section of the silver carp’s gills stained with PAS. The gills comprise the gill arch, the gill filament (primary gill lamellae), and the secondary gill lamellae. The cartilage that is heavily stained by PAS supports the gill filaments

Gills consist of gill arches that bear gill lamellae, both primary and secondary, supported by hyaline cartilage and covered by mucous epithelium. Branchial arteries run longitudinally through the gill arches, sending an afferent arteriole to the gill filaments (Figs. 5, 6).

The squamous epithelial cells of the gills were found to have species-specific ridge patterns on their outer surfaces [87]. These have previously been misidentified as microvilli or stereocillia. Euryhaline species, such as *Tilapia zillii*, have more dense and well-developed ridges than stenohaline species, like *Sarotherodon niloticus*. Freshwater-adapted individuals of *Tilapia zillii, Sarotherodon niloticus, Sarotherodon galflaeus*, and *Tristramella sacra* exhibit slightly swollen surface cells that extend over chloride cell openings. During adaptation to sea water, these ridges rise and become denser, while the cell surface shrinks, revealing the underlying orifices of the chloride cells’ apical crypts. The more euryhaline the species, the less chance there is in the ridge pattern of the cells during the passage from fresh to seawater. This evidence implicates the gill epithelium and the chloride cells in the process of osmoregulation [87].

### Epithelia of the gill arches

The structure comprises multiple layers, including superficial pavement cells, large chloride cells, goblet cells secreting mucus, and basal epithelial cells. Pavement cells exhibit a cuboidal morphology.

**Fig. 7:**
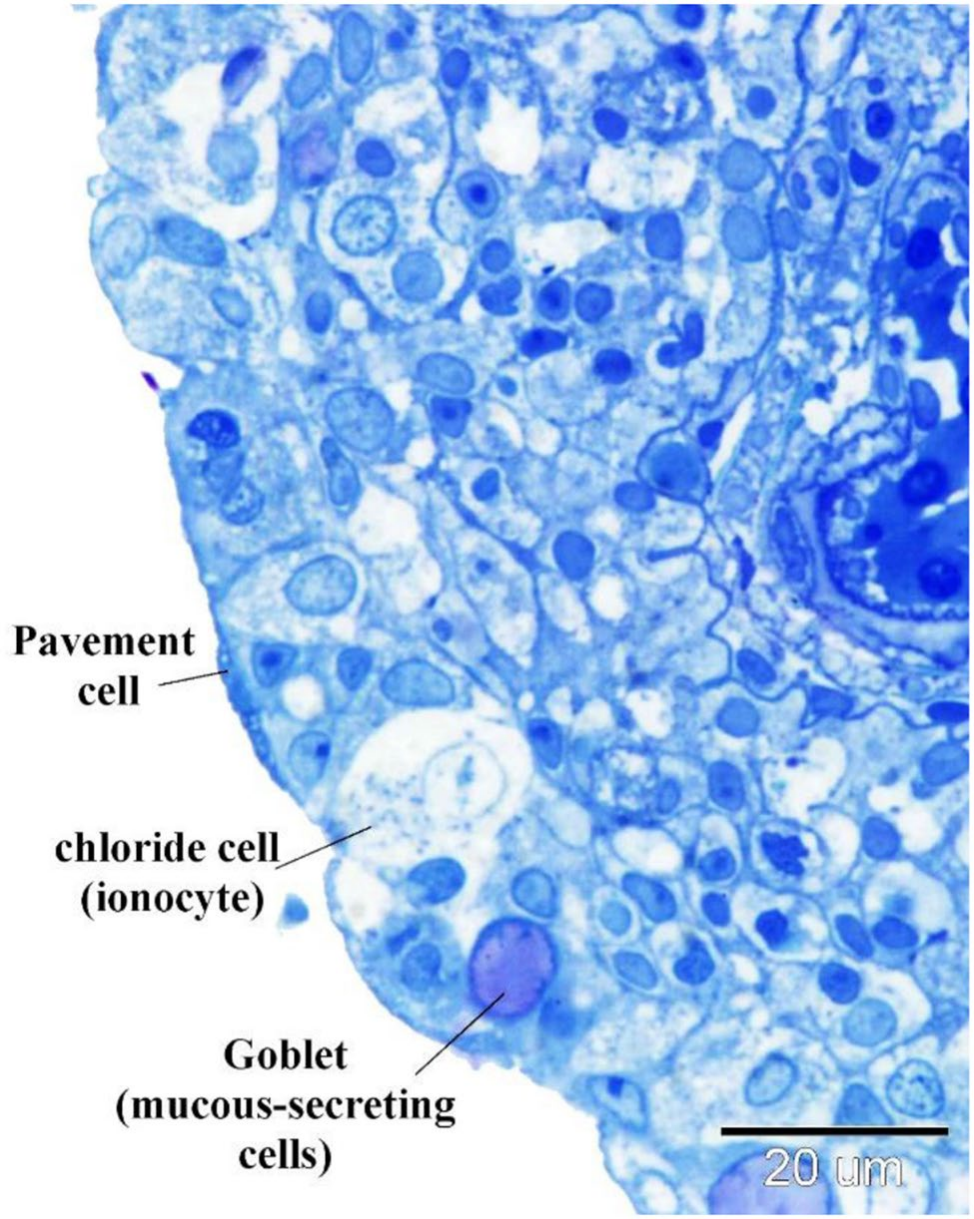
Displays semithin sections of the gill arch of the Ruby-Red-Fin Shark, also known as the Rainbow Shark (*Epalzeorhynchos frenatum*), which belongs to the Teleostei: Cyprinidae family. The epithelia of the gill arch contained goblet cells, chloride cells, and pavement cells. Goblet cells exhibit a high concentration of metachromatic mucus production. The pavement cells had apical striations, which correspond to microvilli. The chloride cells possess cytoplasm with granules and mitochondria

**Fig. 8:**
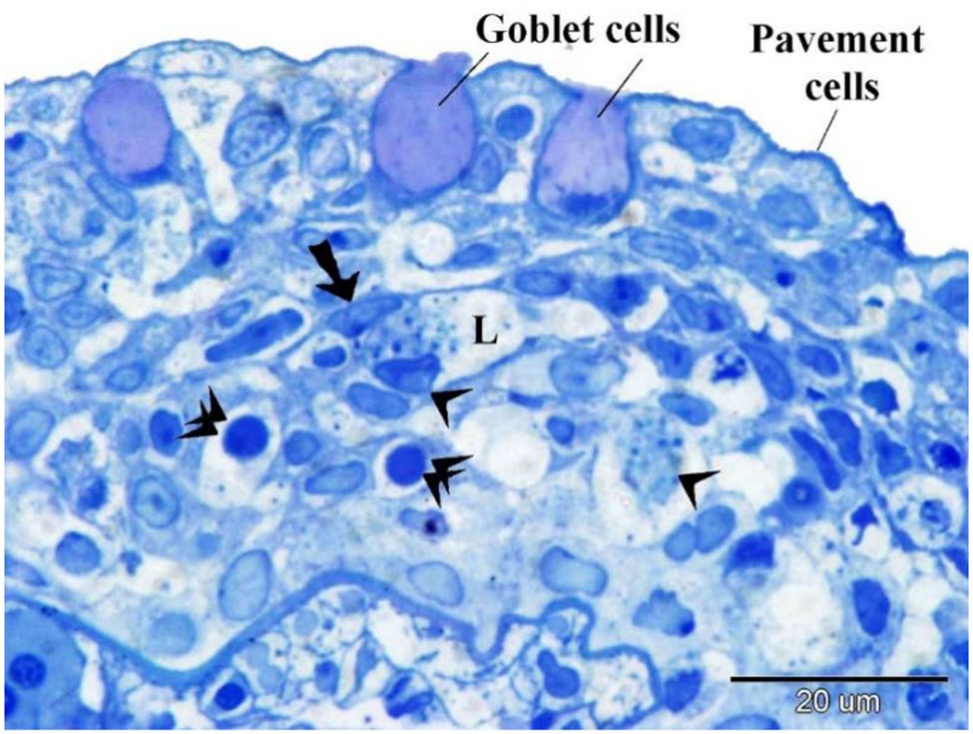
Shows a semithin section of the gill arch of the Ruby-Red-Fin Shark, also known as the Rainbow Shark (*Epalzeorhynchos frenatum*), which belongs to the Teleostei: Cyprinidae family. The gill arch epithelia consists of various cellular kinds. Goblet cells exhibit a high concentration of metachromatic mucus production. The pavement cells exhibited apical striations, which corresponded to microvilli. Telocyte-like cells, indicated by the arrow, comprise the boundary of the lymph space (L). Lymphocytes, indicated by double arrowheads, and granular leukocytes, indicated by arrowheads, are present in the lymphatic space. Note Basal epithelial cells (b)

**Fig. 9:**
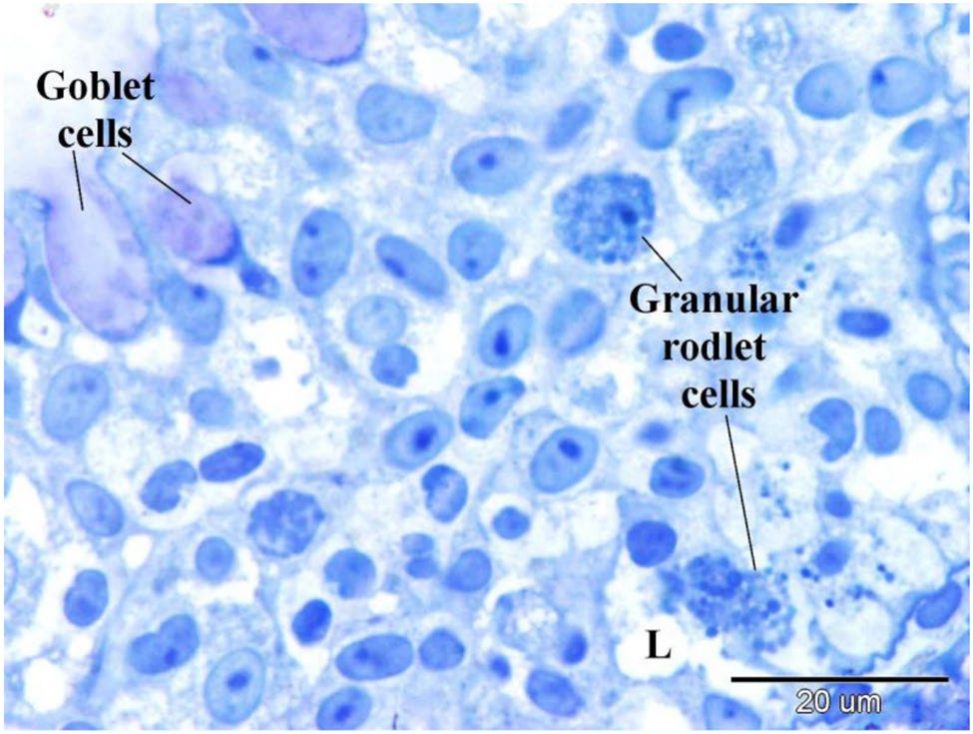
Displays a semithin section of the gill arch of the Ruby-Red-Fin Shark, scientifically known as *Epalzeorhynchos frenatum*, a Teleostei: Cyprinidae family member. The epithelia of the gill arch contained Goblet cells that possess a significant abundance of metachromatic mucus production. Granular rodlet cells are situated within the lymphatic space (L)

Under light microscopy (Fig. 7), the outer surface of pavement cells shows a striated appearance due to micro ridges, which may be microvilli or microplicae. Chloride cells, known as ionocytes, are characterized by their large size and granular cytoplasm rich in mitochondria (Fig. 8). Mucous cells contain vacuoles, observed in H&E staining (Fig. 8), and show metachromatic staining properties with toluidine blue (Figs. 7, 8, 9).

Basal epithelial cells directly contact the basal lamina (Fig. 8) and serve as progenitors for other cell types. The lymph space within the gill arch epithelia contains cells resembling tenocytes, along with immune cells such as lymphocytes and granular leukocytes (Fig. 8), rodlet cells (Fig. 9), and eosinophilic granular cells.

**Fig. 10:**
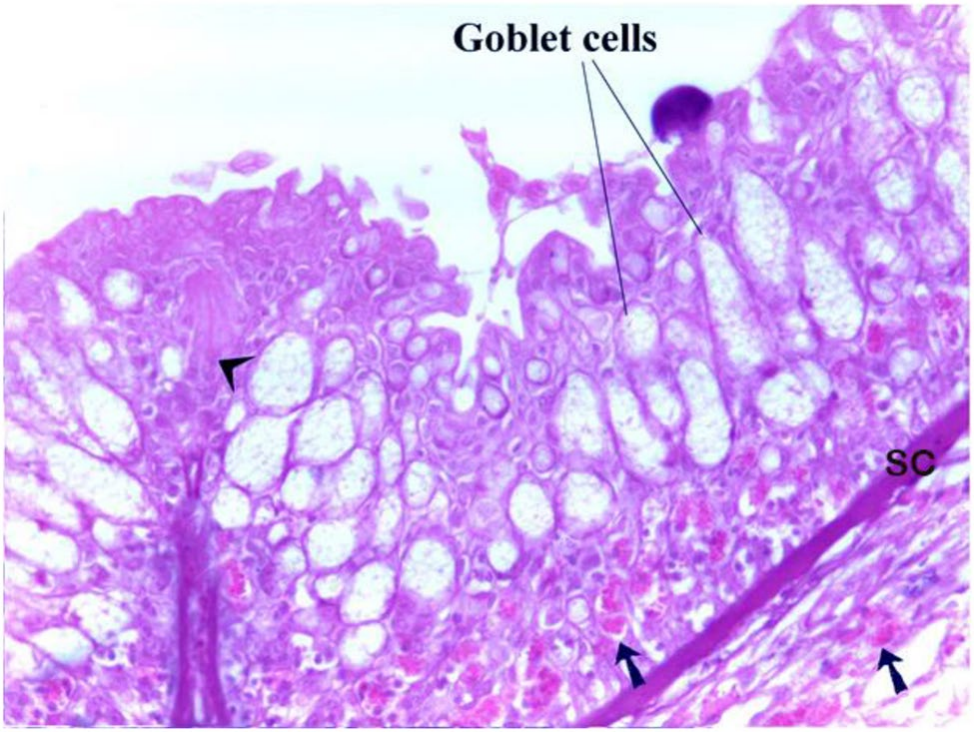
Illustrates the presence of neuromast on the gill arch of a catfish, as indicated by the arrowhead. The goblet cells exhibited pale and vacuolated cytoplasm, with eosinophilic granular cells in the lymph space (arrow). The stratum compactum (SC) is located underneath the basal lamina

**Fig. 11:**
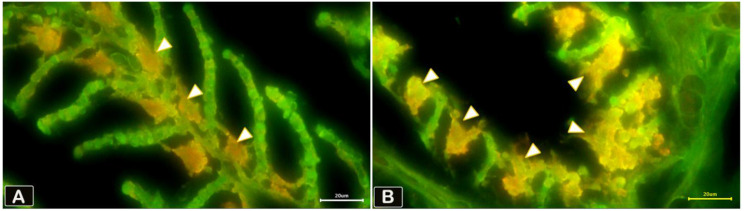
Identification of lysosomal activity of rodlet cells (arrowheads) on paraffin section of the gills of sliver carp using Acridine orange

Gill arches also feature sensory structures called neuromasts, situated on elevated papillae. These structures are composed of sensory hair cells and supporting cells, including stem cells (Figs. 10, 11).

### Filament epithelium

**Fig. 12:**
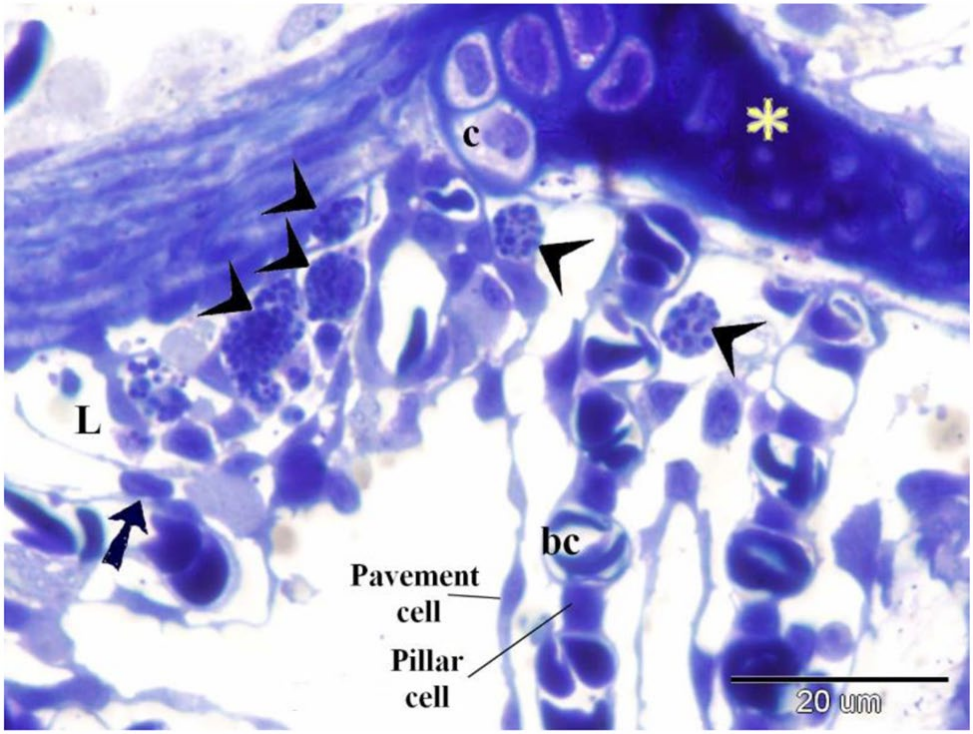
Displays a semithin section of the gill filament and secondary gill lamella of the Ruby-Red-Fin Shark, formally identified as *Epalzeorhynchos frenatum*, a species belonging to the Teleostei: Cyprinidae family. The gill arch is upheld by cartilage containing chondrocytes, situated within a lacuna surrounded by a cartilage matrix (asterisk). Telocyte-like cells (shown by the arrow) are present in the epithelia of the gill arch. These cells are generated within the wall of the lymph space (L). Granular rodlet cells, shown by arrowheads, are situated within the lymphatic space. The secondary gill lamella comprises pillar cells between the blood capillaries. The secondary gill lamella is enveloped by pavement cells

**Fig. 13:**
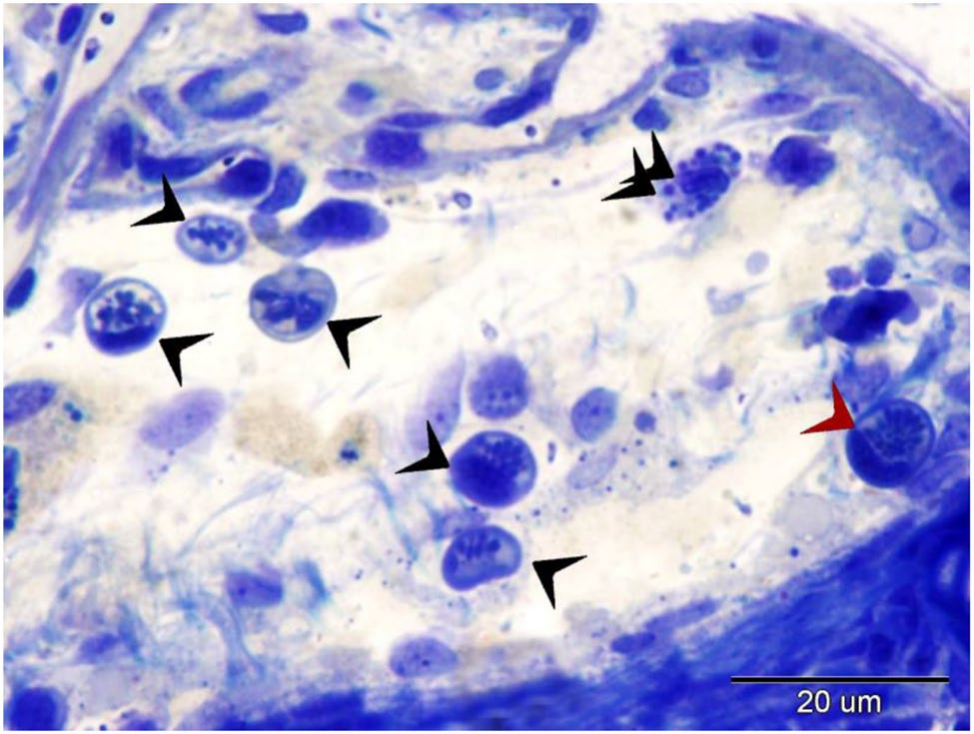
Displays a semithin section of the gill filament of the Ruby-Red-Fin Shark, scientifically known as *Epalzeorhynchos frenatum*. This species is classified under the Teleostei: Cyprinidae family. T. The gill filament comprises granular rodlet cells (shown by double arrowheads) and transitional rodlet cells (indicated by arrowheads), as well as mature rodlet cells (indicated by a red arrowhead)

The gill filament epithelium resembles gill arches, comprising pavement cells, basal epithelial cells, mucous cells, and chloride cells rich in mitochondria [88]. The surface is covered with cuboidal pavement cells, undifferentiated basal cells contact the basal lamina, and intermediate cells occupy the space between [89]. Notably, the interlamellar area of the gill filament epithelium contains numerous large chloride and mucous cells. Both basal and intermediate undifferentiated cells, characterized by a high nucleus-to-cytoplasm ratio, are present in the epithelium of both gill arches and gill filaments. They serve as precursors for specialized cell types (pavement cells and mucous-secreting cells) (Figs. 12, 13).

### Lamellar epithelium

The lamellar epithelium is thinner than the gill filament epithelium and supported by the basal lamina [90]. To enhance gas exchange, it minimizes blood-to-water diffusion distances. Pillar cells, specialized endothelial cells forming blood gaps within the lamellae, are unique to fish gills. Lamellae are arranged like beads on a string. Pavement cells form the outer layer of the epithelium and are characterized as squamous cells. The lamellar epithelium typically lacks mucous and chloride cells [91].

The structure and function of the branchial chloride cell in freshwater fishes [92]. Meanwhile, he stated that the pavement cell is the site of Na+ uptake via channels that are electrically connected to an apical membrane vacuolar H+-ATPase (proton pump). Chloride cells play a critical role in acid-base regulation. Alkalosis increases the surface area of exposed chloride cells, improving base equivalent excretion by increasing the rate of Cl−/HCO3− exchange. In contrast, the chloride cell surface area is reduced during acidosis due to the expansion of adjacent pavement cells. This response decreases the number of functional Cl−/HCO3− exchangers [92].

### Gill arch structure

The gill arch comprises hyaline cartilage in cup-like formations, each consisting of a central cartilaginous core surrounded by a peripheral matrix. Gill filaments extend from these gill arches, each containing a cartilaginous bar covered by an epithelial layer with scattered mucous cells and sparse chloride cells in the interlamellar epithelium. The extending epithelium covers contralateral lamellae, while internal lamellae consist primarily of blood spaces [4].

The gill arches of the European hake are composed of hyaline cartilage arranged in cup-like structures with a central cartilaginous core and peripheral matrix. Gill filaments extend from these gill arches, which are composed of a cartilaginous bar with a peripheral matrix and central core. They are covered by an epithelial layer containing few mucous cells and scattered chloride cells in the interlamellar epithelium. Each gill filament bears several leaf-like secondary lamellae on both sides, lined with epithelial pavement cells, some mucous cells, and pillar cells [17].

Numerous long-gill raker processes from both sides of the gill arch appear in *Clarias gariepinus* [46]. The lateral row gill rakers are present in all gills, including the fifth, while medial row gill rakers are found only in the third and fourth gills, longer in the third. Intermediate row gill rakers are present in all main gills, most developed in the third and weakest in the first.

Particular cells called rodlet cells have been found in freshwater and marine fish [93, 94]. They are displayed in several organs. The respiratory organs, the digestive, vaginal, skin, immunological, circulatory, and skeletal systems, the eye, and the abdominal cavity were among the organs where they were seen [93–95]. Rodlet cells perform various tasks, including osmoregulation and transportation [94, 96], Innate immune cells, and probably leukocyte-derived and sensory function [97]. These cells perform a secretory role [98, 99].

### Neuroepithelial cells

The neuroepithelial cells (NECs) of the fish gill filament are morphologically and functionally most similar to the cells of the neuroepithelial bodies in the lungs of air-breathing vertebrates. In teleosts, neuroepithelial cells are found on the distal half of the filament. In trout, these cells are primarily innervated by non-indolaminergic nerves that pick up sympathetic neurotoxins. The gill filament’s proximal half is made up of isolated NECs that are also innervated by intrinsic indolaminergic neurons. The NECs exhibit serotonin-like immunoreactivity in the granular vesicles packed within the basal soma, as well as processes that surround non-vascular and vascular smooth muscles in the gill filament. Apical processes from neuroepithelial cells occasionally come into contact with water on the surface of the gill filament epithelium [100]. NECS are neurotransmitter-containing chemosensory cells that are diffusely dispersed within a thin epithelial layer of the gill filaments and respiratory lamellae of all gill arches. They are innervated by afferent fibers from the central nervous system. Thus, hypoxic stimulation of gill NECs appears to trigger the production of adaptive cardiorespiratory reflexes that help to maintain O(2) uptake in order to meet metabolic demands [101, 102].

### Dendritic organ in catfish

The dendritic organ is located in the gill arch of catfish. It consists of main stems or smaller branches and end bulbs comprising elastic cartilage, a vascular layer of connective tissue, and an epithelium containing intraepithelial mucous glands. Blood capillaries originate from the vascular layer, penetrate the epithelium, and dilate towards the surface to form respiratory papillae [47, 103]. The dendritic or arborescent organs that project into the suprabranchial chamber and are directly derived from the second and fourth gill arches [104]. All of the epithelia in the gill fans, suprabranchial chamber membrane, and labyrinthine organs contain a series of respiratory islets with transecting capillary flow, indicating that they are derived from the gill filament epithelium. The intraepithelial capillaries extend to the surface, forming respiratory transverse blood capillaries (papillae) [47].

Based on structural and immunohistochemical confirmation, it appears that, in addition to the gills, which are the primary site for O2 chemoreception in fishes, the accessory respiratory organs (ARO) in the air-breathing catfish (*C. gariepinus*) act as a functional system for O2 sensing based on the presence of NEC. NECs have also been found in the specialized respiratory epithelia of some catfish species’ accessory respiratory organs (AROs), including the gills and skin [105, 106].

### Immune system of the gill

The fish gill’s immune functions are closely linked to gill-associated lymphoid tissue (GIALT) and T lymphocytes. GIALT and T lymphocytes can produce immune molecules that aid gill immunity and protect against pathogenic bacteria on mucosal surfaces [107]. In Atlantic salmon (*Salmo salar*) [108], the interbranchial lymphoid tissue (ILT) was recognized. Consequent studies in Atlantic salmon and rainbow trout (*Oncorhynchus mykiss*) demonstrated the presence of T lymphocytes implanted in a meshwork of reticulated epithelial cells in ILT. Only a few immunoglobulin (Ig) M+ lymphocytes were noticed. Still, the structure appears rich in cells expressing major histocompatibility complex (MHC) class II+ cells and IgT transcripts [109, 110]. The ILT must be deliberated as a portion of the gill-associated lymphoid tissue (GIALT), which is defined as one of the four main mucosal immune compartments found in bony fish [111].

### Comparative analysis with mammalian mucosa-associated lymphoid tissue (MALT)

In summary, the structure and function of GALT in fish share both similarities and differences with MALT in mammalian species. Some of the most conserved structures that are GALT analogs to those seen in MALT are secreting mucosa and organized lymphoid tissues. The immune responses in mammalian MALT and fish GALT are quite similar, but differences occur in lymphocytes, M cells, and antimicrobial immunoglobulin production. The function of GALT in fish seems to be part of general mucosal immunity; thus, the main role of this tissue is to fight against the pathogens that mainly invade the host mucosal surfaces. Stimulation of GALT by vaccination has been shown to reduce disease in several common farmed fish species; thus, understanding of GALT abilities is being gradually used for further strategic development in fish aquaculture at the present time. The cells in the GALT provide surveillance against a broad array of intruding pathogens. The cells may be lymphocytes, polymorphonuclear cells, monocytes/macrophages, and myeloid cells. These cells are in the lymphoid population in the gill filaments and interbranchial space. Mechanisms of immune defense such as pathogen trapping, inflammation, antigen processing and presentation, lymphocyte activation, local immunoglobulin production, and NK cell-like activity are also mainly involved in fighting against a wide variety of pathogens in mammalian MALT as well as fish GALT. The types of pathogens encountered by the MALT in any vertebrate species are shaped by the environmental factors and the locations in the bodies of the vertebrates [112–114].

### Gill-associated lymphoid tissue (GALT)

Gill-associated lymphoid tissue (GALT) is a multicomponent system distributed throughout the gills of fish. It consists of the pharyngeal region, including tubular elements of the kidney, and a pair of discrete anterior and posterior caudal regions. The GALT complex is described as the primary interface between the host and the surrounding aquatic environment. This review encompasses the regulation of GALT physiology and the implications of GALT in aquaculture research. GALT consists of a variety of tubular tissue compartments, including granular and glandular sections, and has a number of defined structures that proceed to more distal regions of the gill in a reticulated pattern. GALT is composed of MALT elements, such as macrophages, reticular cells, and plasma cells, arranged within a reticle of crescentic myoepithelial cells. Within the reticle is a labyrinth of capillaries that extends across the gill filaments and is filled with blood in fixed specimens. Two types of benchtop laboratory assays have been developed using isolated and enriched gill leukocytes from rainbow trout as the biological source for investigating the in vitro production of fish immunoglobulin from GALT-associated plasma cells. Reports of the presence of toluidine blue-positive cells within gill tissue in fish of several species have suggested that there may be a link between the presence of GALT and the gill morphology of teleosts.

Gill-associated lymphoid tissue (GALT) is an important primary inductive site of the branchial immune response and has unique immunological and anatomical features, which are likely an adaptation to aquatic life. GALT is the major production area of gill immunoglobulin T (IgT) in the gill repopulation and is also associated with cell surface markers CD3 or CD8, lymphocyte maturation, and differentiation that occurs in the gill. GALT contains high proportions of CD3+ cells among nonadherent leukocytes affected by stress application in the mucosal vaccination site. In the GALT model system, gill fragments transplanted from donor fish were transplanted, leading to plasma cell, CD4+, and secretory IgT plus mucus epithelial cell infiltration into the recipient’s gill tissue and mucus, which is not specific and will not proceed in a time-dependent manner [115–118].

### Function of the gill-associated lymphoid tissue

Gill, as the principal respiratory organ of fish, has been historically linked to respiration and ion regulation. If you are an immunologist like me, you must wonder: what about the immune functions of the gill? Indeed, gill mucosa is rich in immune cells that function not only in immune surveillance but also in maintaining mucosal homeostasis and protecting fish against pathogens and environmental stressors. Its role as an external defense barrier is gaining much attention. This section summarizes the known important immune functions of gill-associated lymphoid tissue and discusses its potential role in mucosal defense against pathogens. The location of the gill allows for continuous antigenic sampling that is conducted primarily by intraepithelial lymphocytes. The teleost gill contains numerous lymphocyte enrichment or induction sites that can effectively guard the gill against pathogenic invasions. It generates a variety of immune functions, including picking up or recognizing pathogens and activating recruited innate leukocytes. In the adaptive immune system, it regulates the activation, maturation, and proliferation of gill leukocytes by releasing cytokines mainly produced by T cells in response to pathogen stimulation. It also plays a role in transporting antigens to peripheral lymphoid tissue for immune memory induction. Activation of mucosal immunity in gills is an important factor in fish mucosal defense and contributes to the overall health and mucosal homeostasis of fish. Fish exposed to live vaccines mainly generate memory T and Ig+ B cells in the gill, so that gill mucosal immunity may also strongly protect fish from pathogenic infections in a contagious manner. Therefore, enhancement of gill mucosal immunity in fish is the most direct way to protect against resistance to mucosal pathogens [44, 112, 119–122].

### Vascular anatomy of the fish gill

Three vascular networks can be found within the gill filament. The arterioarterial (respiratory) pathway comprises lamellae, afferent and efferent segments of the branchial and filamental arteries, and lamellar arterioles. The gill filament has two post-lamellar pathways: interlamellar and nutrient. The interlamellar system is a vast ladder-like network of thin-walled, highly distensible vessels that runs between and parallel to the gill filament’s lamellae and around its afferent and efferent borders. The medial wall of the efferent filamental artery contains short, narrow-bore feeder vessels that supply interlamellar vessels [123–126].

## Conclusion

This review comprehensively examines the gill morphology across various marine and freshwater fish species, highlighting the intricate adaptations to their respective environments and feeding habits. Morphological investigations reveal that the organization of fish gills varies widely. The studies reviewed span diverse fish families, offering detailed insights into the structural variations of gill arches, gill filaments, and gill rakers. We observed significant differences in the arrangement, size, and number of gill rakers between marine and freshwater fishes, reflecting their living and feeding habits. Herbivorous fish like *Siganus luridus* exhibit short gill rakers designed to filter algae efficiently. At the same time, predatory species such as *Pagrus pagrus* possess fewer but longer gill rakers with specialized spines for capturing slippery prey. Omnivorous species like *Boops boops* display intermediate gill raker structures allowing versatile feeding strategies. This indicates that herbivorous species prefer small food particles, whereas carnivorous and omnivorous species tend to consume larger food particles. Microscopic and ultrastructural observations reveal that gill arches are supported by hyaline cartilage and covered by a mucous epithelium rich in various cell types, including pavement cells, chloride cells, and mucous cells. These cells play vital roles in maintaining ion balance, mucus secretion, and forming the protective and functional layers of the gills. SEM studies underscore the presence of complex microstructures such as micro ridges and microplicae on epithelial cells, enhancing the surface area for gas exchange and mucus secretion. The presence of mucous taste buds and immune cells on gill rakers indicates significant surface features contributing to the multifunctionality of gill structures. The dendritic organ in catfish, with its unique structure comprising elastic cartilage, vascular connective tissue, and mucous glands, exemplifies specialized adaptations in freshwater species for enhanced respiratory efficiency. The diverse structural adaptations of gill components across different fish species highlight the evolutionary responses to environmental pressures and dietary needs. This review underscores the importance of gill morphology studies in understanding fish ecology, physiology, and their adaptive mechanisms to diverse habitats. Future research should continue to explore the functional implications of these morphological variations and their role in fish species’ survival and ecological success.

## Data Availability

The datasets used and/or analyzed during the current study are available from the corresponding author on reasonable request.
